# Perioperative Management of a Permanent Pacemaker During Elective Inguinal Hernioplasty Under Spinal Anaesthesia: A Case Report

**DOI:** 10.7759/cureus.112093

**Published:** 2026-07-05

**Authors:** Rajesh Kumar, Gokul Rangan, Sanjay Kumar, Shashi Kant, Rekha Kumari

**Affiliations:** 1 Anaesthesiology and Critical Care, All India Institute of Medical Sciences, Patna, Patna, IND; 2 Anaesthesiology, All India Institute of Medical Sciences, Patna, Patna, IND; 3 Trauma Surgery, All India Institute of Medical Sciences, Patna, Patna, IND

**Keywords:** cardiac implantable electronic device, inguinal hernia, magnet application, perioperative management, permanent pacemaker, spinal anaesthesia

## Abstract

Patients with cardiac implantable electronic devices (CIEDs) are increasingly presenting for non-cardiac surgery, requiring careful perioperative planning to avoid electromagnetic interference, device malfunction, and haemodynamic instability. Although general anaesthesia is frequently used in such patients, regional anaesthesia may offer several advantages when appropriately selected. We report the successful perioperative management of a 75-year-old man with coronary artery disease and a VVIR permanent pacemaker who underwent elective Lichtenstein hernioplasty under spinal anaesthesia. Following a multidisciplinary evaluation, the pacemaker was temporarily converted to asynchronous VOO pacing using magnet application. Continuous electrocardiographic monitoring, avoidance of monopolar electrocautery, availability of external defibrillation, and perioperative cardiology support ensured a safe perioperative period. The intraoperative and postoperative periods were uneventful. This case highlights the importance of structured CIED assessment, individualised pacemaker management, and careful haemodynamic monitoring when neuraxial anaesthesia is considered in patients with permanent pacemakers.

## Introduction

The number of patients living with cardiac implantable electronic devices (CIEDs), including permanent pacemakers and implantable cardioverter-defibrillators, continues to increase worldwide, resulting in a growing number of such patients presenting for non-cardiac surgery [1]. Safe perioperative management requires a thorough understanding of device function, pacing dependency, battery status, and the potential effects of electromagnetic interference on device performance [2].

Electromagnetic interference generated by electrosurgical equipment may inhibit pacing, trigger inappropriate device behaviour, or rarely result in device malfunction [3]. Consequently, current guidelines recommend preoperative device interrogation, assessment of pacing dependency, consideration of magnet application or device reprogramming, and close collaboration with cardiology and device specialists to minimise perioperative risk [1,3].

Regional anaesthesia may offer several advantages in selected patients with cardiovascular disease by avoiding airway manipulation, reducing the stress response to surgery, and providing effective perioperative analgesia. However, neuraxial anaesthesia can result in sympathetic blockade, leading to hypotension and bradycardia, which may be poorly tolerated in patients with limited cardiovascular reserve [4].

We report the successful perioperative management of a patient with a permanent pacemaker undergoing elective inguinal hernioplasty under low-dose spinal anaesthesia following temporary conversion to asynchronous pacing using magnet application.

## Case presentation

A 75-year-old male patient weighing 68 kg was scheduled for elective left-sided Lichtenstein hernioplasty for a reducible indirect inguinal hernia. His medical history was significant for coronary artery disease, recurrent palpitations, syncopal episodes, and a previous cardiac arrest that required cardiopulmonary resuscitation.

Following a cardiology evaluation, a St. Jude permanent pacemaker was implanted. The patient remained on regular follow-up and was receiving oral metoprolol (100 mg twice daily) and oral aspirin (75 mg once daily).

Preoperative pacemaker interrogation performed six months before surgery demonstrated VVIR mode, output voltage of 2.0 V, battery current of 10 μA, lower rate limit of 60 beats/minute, and battery longevity greater than 90%, with no recorded atrial tachyarrhythmias. Electrocardiography showed a normal sinus rhythm with an intrinsic heart rate exceeding 90 beats/min. The patient was classified as New York Heart Association (NYHA) functional class II, with an exercise capacity below four metabolic equivalents (METs).

Routine laboratory investigations, including complete blood count, renal function tests, liver function tests, coagulation profile, serum electrolytes, and blood glucose levels, were within normal limits.

Following a multidisciplinary discussion involving the anaesthesiology, cardiology, and surgical teams, low-dose spinal anaesthesia was selected. General anaesthesia was used as a backup option. A pacemaker technician was available throughout the procedure and in the immediate postoperative period.

On the day of surgery, continuous electrocardiogram (ECG) monitoring was established. During the preoperative cardiology evaluation, the expected magnet response was confirmed in accordance with the device manufacturer's specifications. A magnet was then placed over the pacemaker generator, resulting in the temporary conversion of the device from VVIR mode to asynchronous VOO pacing, as anticipated. External defibrillation equipment and emergency pacing facilities were immediately available. The surgical team was advised to avoid monopolar electrocautery and to use bipolar cautery only if required.

Upon arrival at the operating room, standard American Society of Anesthesiologists (ASA) monitoring was performed. The baseline blood pressure was 128/72 mmHg, the heart rate was 92 beats/minute, and the oxygen saturation was 99% on room air. Intravenous access was secured, and crystalloid co-loading was initiated before the procedure.

A subarachnoid block was performed in the sitting position at the L3-L4 interspace using a 25-gauge Quincke needle. Hyperbaric bupivacaine 0.5% (2.5 mL) combined with fentanyl 25 μg (0.5 mL) was administered to the patient. Sensory blockade of the T10 dermatome was achieved within six minutes.

The surgery was uneventful. No episodes of hypotension, bradycardia, arrhythmia, oxygen desaturation, or pacemaker malfunction were observed. The haemodynamic parameters remained stable throughout the procedure.

Following the completion of the surgery, the patient was transferred to the recovery area under continuous ECG monitoring. Removal of the magnet restored the pacemaker to the original VVIR mode. Postoperative observation over 24 hours revealed a stable cardiac rhythm and haemodynamics. The patient was discharged without any complications.

## Discussion

This case demonstrates that spinal anaesthesia can be safely administered in selected patients with permanent pacemakers when contemporary CIED management principles are followed.

The first step in perioperative management is the identification of device type, pacing mode, battery status, and pacing dependency. Current recommendations emphasise recent device interrogation and individualised perioperative planning before surgery [5]. Our patient underwent preoperative cardiology assessment and device evaluation, which confirmed satisfactory pacemaker function and adequate battery reserve.

Electromagnetic interference remains a major concern during surgery in patients with CIEDs. Monopolar electrocautery can interfere with sensing circuits and may result in pacing inhibition or inappropriate device behaviour. Consequently, bipolar cautery is preferred whenever feasible, and the surgical field should be kept as far from the device as possible. Recent evidence continues to highlight the importance of understanding device-specific responses and the utility of magnet application during perioperative management [6].

Magnet application is a widely accepted strategy for temporary perioperative pacemaker management. However, the response to magnet placement varies according to device manufacturer and programming settings; therefore, preoperative verification of magnet response is essential [6]. In our patient, the magnet application successfully converted the device from VVIR mode to asynchronous VOO pacing, providing predictable rhythm control throughout the procedure.

Low-dose spinal anaesthesia combined with intrathecal opioids may provide adequate surgical anaesthesia while minimising the extent of sympathetic blockade. Ben-David et al. demonstrated that intrathecal low-dose bupivacaine with fentanyl provides effective anaesthesia with improved haemodynamic stability for cesarean delivery [7]. A similar strategy was adopted in our patient to reduce the risk of significant hypotension and bradycardia associated with conventional spinal anaesthesia.

Another important aspect of management is comprehensive cardiovascular risk assessment and multidisciplinary perioperative planning. The 2014 American College of Cardiology (ACC)/American Heart Association (AHA) guidelines emphasise individualised perioperative evaluation, optimisation of cardiovascular status, and coordinated decision-making among anaesthesiologists, surgeons, and cardiologists for patients with significant cardiovascular disease undergoing non-cardiac surgery [8].

Rozner further highlighted the importance of understanding pacemaker function, perioperative device management, and appropriate strategies to prevent device-related complications during anaesthesia [9]. Adherence to these principles contributed to the uneventful perioperative course observed in our patient.

The novelty of the present report lies in the successful use of low-dose spinal anaesthesia combined with magnet-assisted asynchronous pacing in an elderly patient with coronary artery disease and a permanent pacemaker undergoing elective inguinal hernioplasty. Although perioperative management of CIEDs has been widely discussed, reports describing neuraxial anaesthesia with temporary magnet-guided pacing remain relatively limited.

This case highlights the importance of multidisciplinary planning, individualised pacemaker management, avoidance of electromagnetic interference, and continuous haemodynamic monitoring in achieving favourable perioperative outcomes.

## Conclusions

Patients with permanent pacemakers can safely undergo non-cardiac surgery under spinal anaesthesia when careful perioperative assessment and device management strategies are employed. Preoperative device interrogation, appropriate use of magnet application, avoidance of electromagnetic interference, and continuous haemodynamic monitoring are essential components of care. This case highlights that low-dose spinal anaesthesia may be a safe and effective option for selected patients with permanent pacemakers undergoing lower abdominal surgery when supported by multidisciplinary planning and adherence to contemporary CIED management guidelines.
